# Effect of wearing a dorsiflexion assist orthosis on mobility, perceived fatigue and exertion during the six-minute walk test in people with multiple sclerosis: a randomised cross-over protocol

**DOI:** 10.1186/1471-2377-12-27

**Published:** 2012-05-25

**Authors:** James McLoughlin, Christopher Barr, Daina Sturnieks, Stephen Lord, Maria Crotty

**Affiliations:** 1 Flinders University South Australia, GPO Box 2100, Adelaide 5001, Australia; 2 Neuroscience Research Australia, PO Box 1165, Randwick, NSW 2031, Australia; 3 University of New South Wales, Sydney, NSW 2052, Australia

**Keywords:** Multiple sclerosis, Fatigue, Gait

## Abstract

**Background:**

Fatigue in combination with gait and balance impairments can severely limit daily activities in people with multiple sclerosis (PWMS). Generalised fatigue has a major impact on walking ability, with moderately disabled PWMS experiencing difficulty in walking extended distances. Localised motor fatigue in the ankle dorsiflexors can lead to foot drop, further reducing functional ambulation. The aim of this study is to evaluate the effect of a simple dynamic dorsiflexion assist orthosis on walking-induced fatigue, gait, balance and functional mobility in PWMS.

**Methods:**

A randomised cross-over trial will be conducted with 40 community dwelling PWMS with mild to moderate mobility disability. Participants will initially be screened for disease severity, balance, strength, depression and fatigue at the South Australian Motion Analysis Centre. On two non-consecutive occasions, within two weeks, participants will undergo either the 6-minute walk test (6MWT) or the 6MWT while wearing a dorsiflexion ankle orthosis (with a randomised condition order). Distance walked, perceived exertion, perceived fatigue and the physiological cost of walking (the primary outcome measures) will be compared between the two walking conditions. Additional pre- and post-6MWT assessments for the two conditions will include tests of strength, reaction time, gait and balance.

**Discussion:**

This study will increase our understanding of motor fatigue on gait and balance control in PWMS and elucidate the effect of a Dynamic Ankle Orthosis on fatigue-related balance and gait in PWMS. It will also examine relationships between mobility and balance performance with perceived fatigue levels in this group.

**Trial Registration Number:**

ACTRN12612000218897

## Background

People with Multiple Sclerosis (PWMS) have difficulties with gait and balance that can significantly limit activities of daily living and may increase the risk of falls [1]. Disruption to central nervous system integration of proprioception, visual and vestibular sensory information are all likely to contribute to balance problems and fall risk in this population [1-3]. Perceived levels of fatigue are also associated with balance problems and falls in PWMS [4], while exercise-induced fatigue has been shown to reduce balance control in a backward leaning task [5].

Changes to gait can be detected early in the disease course, even in PWMS without any mobility limitations [6]. In fact, early gait and balance changes can be detected in PWMS who have no pyramidal signs or clinical disability [7]. These changes may be, in part, due to lower limb motor fatigue, which is commonly reported in PWMS and appears to worsen with increasing walking distance [8]. A previous study showed no significant kinematic variability in gait with fatigue in a mildly disabled group of PWMS [9]. However, fatigue related changes in gait variability may become apparent in PWMS with more severe disability levels. As with generalized fatigue in MS, our current understanding of motor fatigue is limited. Ankle dorsifexors are particularly susceptible to the effects of motor fatigue, with both central and peripheral neuromuscular alterations contributing to fatigue-related weakness [10].

In rehabilitation, treatments addressing dorsiflexion weakness during gait can include specific dorsiflexor exercise and/or prescription of an Ankle Foot Orthoses [11]. To date, studies have been unable to indicate the effectiveness of these orthoses in improving ambulation in PWMS [12]. Static and dynamic ankle foot orthoses have been shown to improve static balance, but impair dynamic balance when walking [13]. The Dorsiflexor Assist Orthosis (DAO) (Foot Up, Ossur©) differs from standard ankle orthoses as it dynamically assists the foot into dorsifexion with an elastic strap without affecting the sensory input of the foot inside the shoe.

Moderately disabled ambulatory PWMS who have mobility difficulties that impact on their ability to undertake daily activities often seek outpatient or community physiotherapy with the aim of improving or maintaining mobility levels, but unfortunately, there is limited evidence to inform clinicians with regard to best practice in this area. The aim of this study, therefore, is to evaluate the effect of a possible therapeutic device (i.e. a DAO) on the six-minute walk test (6MWT), walking efficiency and perceived levels of fatigue and exertion. The 6MWT was selected as the fatiguing condition as it has been evaluated in PWMS as a measure of functional capacity and because PWMS have reduced 6MWT distance when compared with controls [14]. In addition, we anticipate that in a moderately disabled group, the 6MWT will induce fatigue and changes to gait and balance control. We will therefore examine any deterioration in function by in gait patterns, balance and tests of motor function following walking the 6MWT with and without the DAO.

## Methods

### Design

**Figure 1:**
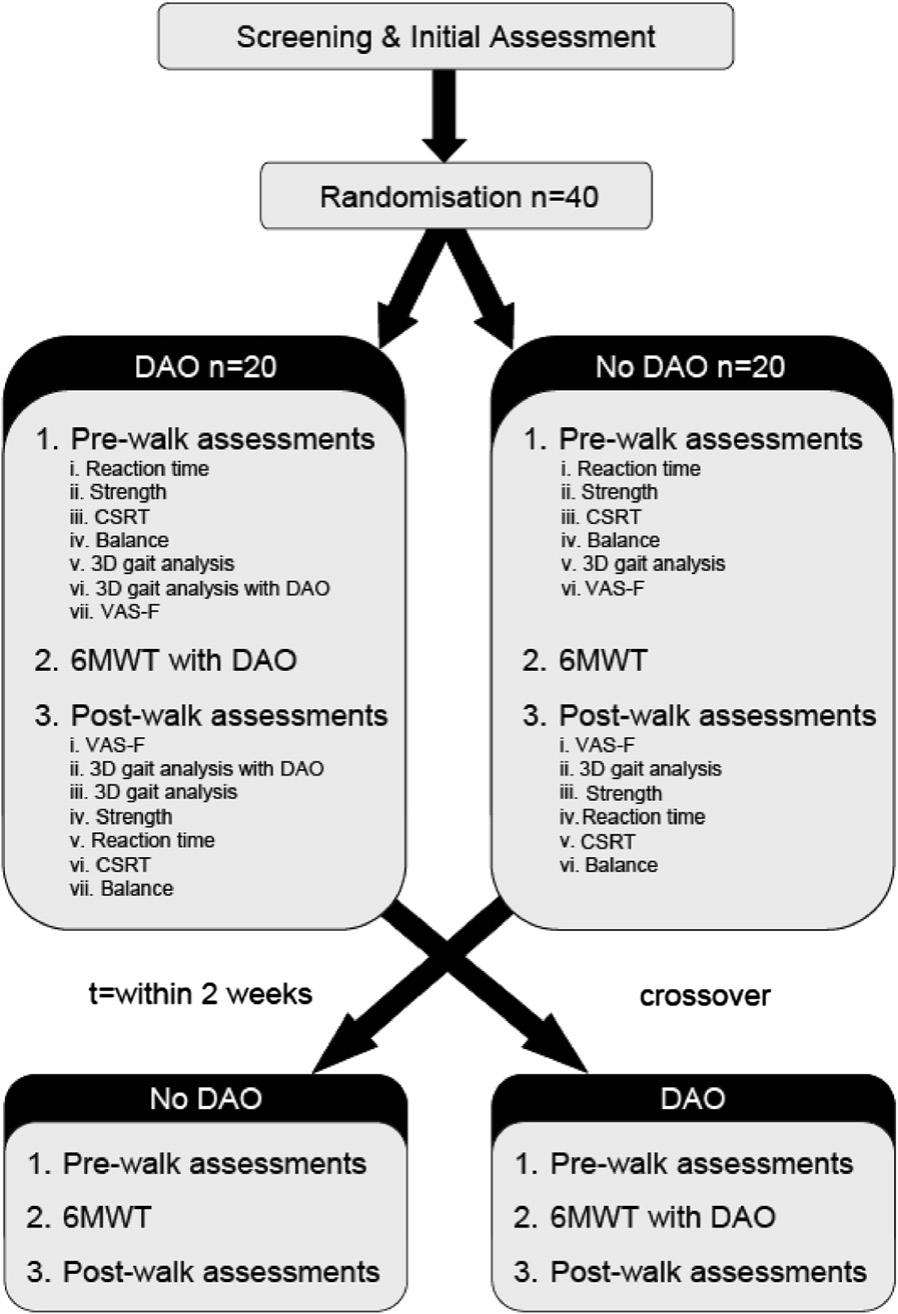
Randomised cross-over trial design.

A randomised cross-over trial will be conducted with 40 community-dwelling PWMS (Figure 1).

### Participants

Participants will be recruited from the MS Society of South Australia, the multidisciplinary MS Clinic at Repatriation General Hospital (RGH) and from private physiotherapy clinics in South Australia. To be included, participants must: (i) have a definite diagnosis of MS; (ii) have mild to moderate difficulty in mobility with an Expanded Disability Status Scale (EDSS) score of 3.0-6.0; (iii) be able to walk for 6 minutes unaided or with the aid of a walking stick. Participants will be excluded if they: (i) have had an exacerbation or relapse of MS within the past 3 months; (ii) use medication prescribed for fatigue or mobility such as Amantadine, Modafinil or Fampridine; (iii) have significant cardiac or respiratory disease; (iv) suffer from severe depression, or (v) have arthritis, fibromyalgia or pain that severely limits walking. All participants will provide informed written consent prior to involvement in the study. The Repatriation General Hospital Research and Ethics Committee have approved the study protocol, registration number EC00191.

### Setting/location

Assessments will take place at the South Australian Movement Analysis Centre at the Repatriation General Hospital, Adelaide.

### Screening and initial assessments

Participants will initially be screened for disease severity with the EDSS by a certified Neurostatus investigator (http://www.neurostatus.net). Additional initial assessments will include: (i) tests of vision, strength, balance, reaction time and sensation [15]; (ii) measurements of height and weight to calculate Body Mass Index (BMI); (iii) the Fatigue Severity Scale (FSS) [16]; (iv) the Modified Fatigue Impact Scale (MFIS) [17,18]; (v) the Beck Depression Inventory –Fast Screen (BDI-FS) [19]; and (vi) the Multiple Sclerosis Walking Scale (MSWS-12) [20,21].

### The walking protocol and Pre- and post-walk assessments

Following initial screening and assessments, participants will be randomised with respect to condition order (i.e. 6MWT with or without DAO), and subsequently undertake the baseline pre-walk assessments. Immediately following the 6MWT, participants will undertake the post-walk assessments. The pre- and post-walk assessments include tests of strength, simple reaction time, choice stepping reaction time, standing balance, and gait analysis, as described below and presented in Figure 1. Participants will return to the laboratory within 2 weeks to complete the other 6MWT condition and associated pre- and post-walk assessments.

#### Walking protocol & perceived exertion

Participants will wear a Polar heart rate monitor during each walking condition. Participants will be seated at rest for 2 minutes to determine a baseline heart rate. We will use a modified 6MWT version from the American Thoracic Society script with the aim of maximising effort and inducing fatigue [15]. Participants will be instructed to walk back and forth along a ten-metre walkway as fast as possible over the six-minute testing period with the instructions “walk as fast as you comfortably can, bearing in mind that you will be walking for 6 minutes”. Participants will be permitted to use assistive devices (i.e. canes) if required. Distance walked and perceived exertion will be recorded each minute using the 10-point Modified Borg Rating of Perceived Exertion (RPE) scale [16], and heart rate (beats/min) will be automatically recorded at 5-second intervals. Physiological Cost Index of walking will be calculated by the formula;

(1) $$\left(\text{average 6MWT HR }–\text{ average resting HR}\right)/\text{ walk speed}\text{,}$$

where heart rate is measured in beats/min and walk speed measured in m/min [17].

#### Perceived fatigue levels

Participants will be asked to indicate their perceived fatigue levels will be measured using the Visual Analogue Scale for Fatigue (VAS-F) [18] immediately before and after the 6MWT.

#### 3D Gait analysis

3D gait analysis will be conducted using an 8 camera Vicon MX3 system (Vicon, Oxford, UK) with 4 AMTI (AMTI, Watertown, MA) force plates and Helen Hayes marker set [19]. Spatiotemporal data and kinematic and kinetic data for the hip knee and ankle will be collected over a minimum of 6 walking trials with and without the DAO. Data will be collected using Vicon Nexus software (v1.4) and processed with Matlab (v7.0). Sagittal plane angles at the ankle are of particular interest, to examine effects of the DAO, in addition to knee and hip angles to identify potential compensations for DAO effects at the ankle.

#### Strength

Knee extension and ankle dorsiflexion strength will be determined as isometric force (kg) produced, measured with a strain gauge linear to direction of force production, normalised to body mass. Strength tests will be conducted in a seated position. For knee strength testing, the hip and knee will be positioned in 90 degrees of flexion. The test of ankle strength will be conducted with the ankle at 30 degrees of plantarflexion. Each strength test will be repeated 3 times with sufficient rest in between, and the maximum score recorded [20].

#### Simple reaction time

Participants will be seated in a chair with ceiling lights dimmed. Simple reaction time will be measured in milliseconds using a handheld electronic timer, with participants seated and using a light as the stimulus and a finger-press (hand reaction time) or a foot-press (foot reaction time) as the response. Five practice trials will be followed by an additional 10 trials, for which reaction time in milliseconds will be recorded for each trial and averaged [20].

#### Choice stepping reaction time (CSRT)

The CSRT device consists of a floor-mounted mat that contains six plates (32 X 13 cm): two base plates on which the participant stands and four target plates. A computer monitor displays a corresponding image of the mat. One of the stepping plates on the computer image light up in a random order. The participant will be instructed to step on to the corresponding illuminated plate as quickly as possible, using their left foot for the targets to the left and to the front left, and their right for the targets to their right and front right. Reaction times for (i) time to lift the foot from the base plate and (ii) time to land the foot on target plate will be recorded. Each plate will be presented 5 times, and sufficient time will be given between each trial to re-establish balance and minimise fatigue [21,22].

#### Standing balance

Standing balance will be measured while participants stand on two AMTI force plates. Postural position of C7 and sacrum will be measured using reflective markers and an 8-camera Vicon© MX3 system. The participant will stand for 30 seconds under 4 conditions: (i) eyes open feet apart; (ii) eyes open feet together; (iii) eyes closed feet apart; and (iv) eyes closed feet together. Each condition will be repeated with the 8 trials presented in a randomized order.

### Primary outcome measures

The primary outcome measures are walking distance, physiological cost index and perceived fatigue and exertion during the 6MWT.

### Secondary outcome measures

The secondary outcome measures are hand and foot simple reaction time, choice stepping reaction time, knee extension and ankle dorsiflexion strength, postural sway with feet together and apart and the spatio-temporal, kinematic and kinetic gait parameters.

### Sample size calculations

We used the “Inequality Tests for Two Means in a 2x2 Cross-Over Design using Differences” module from the Power Analysis and Sample Size (PASS) software [18] for estimating the sample size required for the primary outcome measure of 6MWT distance. The following parameters were entered into the calculation: power = 0.8; alpha = 0.05; N(total sample size) = 20,30,40; mean difference between groups at retest = .50,100,150. Standard deviation measures were estimated from previous studies [11]. The difference in 6MWT distance was based on the rationale that a clinically meaningful increase in metres walked by MS patients in this test is 100 m (i.e. the difference reported between Mild MS and Moderate MS [23]). These analyses indicated that a sample size of 35 would be appropriate for the study design. An additional 5 participants will be recruited to allow for possible dropouts.

### Statistical analysis

Initially we will examine the frequency distributions, percentages and calculate means and standard deviations (mean +/−SD) for the outcome measures. Repeated measures analysis of variance (ANOVA) will be used to determine differences in the outcome measures with time and DAO condition as factors. Finally, we will examine associations between 6MWT and measures of fatigue (FSS, MFIS VAS-F & Borg RPE), EDSS scores and Postural Sway data with Pearson correlations. The data will be analysed with the Statistical Package for Social Science (SPSS) software.

We will adjust for other possible confounding variables by using a covariance analysis. Multiple regression analysis will be used to examine significant, independent predictors of 6MWT distance.

## Discussion

This study will examine the use of a novel ankle dorsiflexion assisting device (DAO) on walking performance in people with moderate MS and determine potential beneficial effects on walking induced changes in fatigue, gait patterns, strength, balance and reaction time. Ambulation distance has historically been a key measure of functional status and disability in PWMS [24]. A recent multidisciplinary consensus conference in 2007, with the Consortium of MS Centres in the USA, agreed that rehabilitation professionals need to improve their understanding of gait and fatigue outcome measures used in MS rehabilitation [22].

While it is known that PWMS have reduced mobility, impaired balance and higher fatigue levels compared with healthy controls, the complex interaction between these factors is not well understood. PWMS have a reduced 6MWT performance compared to healthy controls, and it is important to identify the changes in fatigue, gait, balance and motor function that result in this reduced mobility as this may identify key modifiable targets for rehabilitation. These targets may include either exercise and/or timely use of specific orthoses to maximise functional mobility while reducing the risk of falls. We believe this detailed study design will allow us to further determine what kinematic and kinetic changes are associated with prolonged walking in PWMS, providing a more detailed understanding of complex movement dysfunctions such as spasticity and localised motor fatigue. This may also help in designing individualised orthoses aimed at maximising mobility without compromising balance control. In addition we hope to deepen our knowledge of perceived fatigue and physiological performance associated with mobility.

It is likely a rehabilitation approach that uses exercises that address aspects of strength and skilled recruitment of muscles involved in walking and dynamic balance may need to be combined with individually prescribed orthoses in order to maximise mobility for PWMS. Our ability to analyse gait and balance becomes increasingly important when considering recent therapeutic advancements that have been shown to reduce clinical progression, improve disability and increase ambulation levels [25,26]. This is an important step for future research directed towards evaluating targeted interventions for improving balance and mobility and reducing fatigue in PWMS.

## Abbreviations

PWMS: People with Multiple Sclerosis; 6MWT: Six-Minute Walk Test; DAO: Dynamic Ankle Orthosis; RGH: Repatriation General Hospital; EDSS: Expanded Disability Status Scale; MS: Multiple Sclerosis; BMI: Body Mass Index; FSS: Fatigue Severity Scale; MFIS: Modifies Fatigue Impact Scale; BDI-FS: Beck Depression Inventory- Fast Scale; MSWS-12: Multiple Sclerosis Walking Scale; RPE: Rating of Perceived Exertion; VAS-F: Visual Analogue Scale for Fatigue; CSRT: Choice Stepping Reaction Time; PASS: Power Analysis and Sample Size; ANOVA: Analysis of Variance.

## Competing Interests

The Physiological Profile Assessment (FallScreen) is commercially available through Neuroscience Research Australia.

## Authors’ contributions

JMcL SL DS and MC conceived the idea. All authors obtained funding for the study. All authors contributed to the design and development of the trial protocol. JMcL, CB& DS drafted the manuscript. All authors critically reviewed the manuscript and approved the final manuscript.

## Pre-publication history

The pre-publication history for this paper can be accessed here:

http://www.biomedcentral.com/1471-2377/12/27/prepub
